# Disentangling the Frames, the State of Research on the Alphavirus 6K and TF Proteins

**DOI:** 10.3390/v9080228

**Published:** 2017-08-18

**Authors:** Jolene Ramsey, Suchetana Mukhopadhyay

**Affiliations:** Department of Biology at Indiana University, Bloomington, IN 47405, USA; jolramse@indiana.edu

**Keywords:** alphavirus, 6K, transframe, frameshifting, budding

## Abstract

For 30 years it was thought the alphavirus *6K* gene encoded a single 6 kDa protein. However, through a bioinformatics search 10 years ago, it was discovered that there is a frameshifting event and two proteins, 6K and transframe (TF), are translated from the *6K* gene. Thus, many functions attributed to the 6K protein needed reevaluation to determine if they properly belong to 6K, TF, or both proteins. In this mini-review, we reevaluate the past research on 6K and put those results in context where there are two proteins, 6K and TF, instead of one. Additionally, we discuss the most cogent outstanding questions for 6K and TF research, including their collective importance in alphavirus budding and their potential importance in disease based on the latest virulence data.

## 1. Introduction

For 20 years, alphavirus researchers studied a 6 kilodalton protein aptly named 6K. This protein was identified to play a role in virus exit and was also determined to function as an ion channel. The 6K protein was not required for virus infection. In 2008, a bioinformatics team discovered that what had been termed the 6K protein was actually two proteins. A heptanucleotide slippery sequence within the *6K* gene directs ribosomal frameshifting and the consequent translation in the (−1) open reading frame produces the transframe protein, or TF. The 6K and TF proteins have identical N-termini, but unique C-termini. When TF was identified, the accumulated literature was reevaluated to disentangle the results that applied specifically to 6K, to TF, or to both. Within the last nine years, several reports have emerged addressing the specific activities of TF and 6K. Here we address the current state of our knowledge about 6K and TF and explore the most cogent questions remaining to determine their function in an alphavirus infection.

## 2. The Assembly-Centric Alphavirus Lifecycle

Classified within the Togaviridae family, the Alphaviruses are enveloped particles transmitted by arthropods ([1] and references therein). Virions are ~70 nm in diameter. The viral lipid bilayer contains 240 copies each of two envelope glycoproteins, E2 and E1, forming an exterior proteinaceous shell. Beneath the bilayer is a second protein shell called the nucleocapsid core. Both the exterior glycoproteins and the interior core have T = 4 icosahedral symmetry. The nucleocapsid core protein surrounds a single copy of the single-stranded, capped, and polyadenylated ~12 kilobase RNA genome. Depending on the alphavirus species, the viral proteins E3, 6K, or TF may also be present in the virion, but these are not required for the particle to be infectious [1]. Host molecules are generally absent in wild-type (WT) virus, though ribosomal components have been shown to be packaged within the core [2].

As arboviruses, alphaviruses are generally transmitted in the environment via an arthropod vector (typically a mosquito) to vertebrate hosts which can include birds, humans, and other mammals [3]. In the laboratory, alphaviruses can replicate efficiently in small animal models and various host-derived cell lines. Most animals experience a high-morbidity, low-mortality infection, but this varies by alphavirus species. At the cellular level, arthropod host cells typically experience a persistent infection, while vertebrate cells expire after an acute infection [4].

To infect host cells, the E2 glycoprotein on the virion binds to host cell surface receptors [1]. Following endocytosis, a low-pH mediated conformational change in E1 activates it to mediate fusion between the host membrane and virus membrane. Translation begins immediately upon delivering the positive-sense RNA genome into the cytoplasm of a host cell. The first open reading frame codes for the nonstructural proteins. The four nonstructural proteins function in RNA replication and modulation of the host response. For an in-depth description of RNA replication functions, we refer readers to a recent review by Rupp and colleagues [5]. A review by Fros and Pijlman describes our current understanding of the interplay between virus takeover and host cell responses [6]. The host cell responses are countered in a vertebrate cell infection by a rapid shutoff of host cell protein production that can be mediated by nonstructural protein 2 or the structural protein capsid [7,8,9].

**Figure 1:**
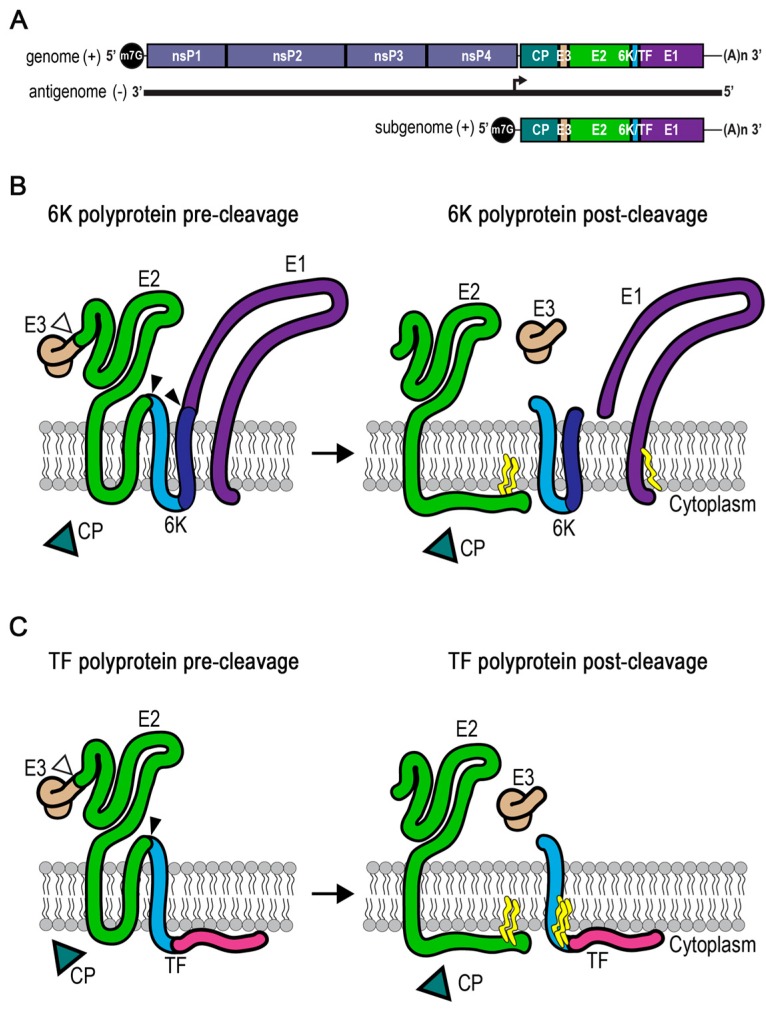
Structural polyprotein membrane topology and processing. (**A**) The positive-sense RNA alphavirus genome has two open reading frames. At the 5’ end is the methyl-7-guanosine cap (m7G), followed by the nonstructural protein (nsP) and structural protein open reading frames, and the 3’ poly A tail. Nonstructural proteins are translated directly from the genome. During replication, the minus-sense antigenome is used as the template for producing the subgenomic RNA from an internal subgenomic promoter (arrow). Structural proteins are translated from the subgenomic transcript. (**B**,**C**) Pre-cleavage structural polyprotein conformation in the endoplasmic reticulum (ER) membrane. Capsid (CP) is first released as a soluble protein in the cytoplasm. The N-terminus of E3 contains a signal sequence that directs translocation across the membrane. Open arrows mark the furin cleavage site between E3 and E2, which occurs in the *trans*-Golgi network. The remaining envelope proteins are threaded through the membrane with insertion of transmembrane domains. Closed arrows mark signalase cleavage sites that are used in the ER. Post-cleavage conformations include palmitoylation sites (yellow). (**B**) The 6K polyprotein is the majority translation product. (**C**) The TF form of the polyprotein is the minority product resulting from frameshifting in the *6K* gene, and does not include E1.

The virus structural proteins are all translated as a polyprotein from the second open reading frame on a subgenomic RNA transcript [1] (Figure 1A). To understand the consequences of translating 6K versus TF, it is necessary to understand how the structural proteins are translated, translocated into the endoplasmic reticulum (ER), cleaved, and then oligomerized to form functional assembly components. The capsid protein is translated, folded in the cytoplasm, and then cleaved in *cis* via its chymotrypsin-like domain from the rest of the polyprotein [10,11,12]. The capsid protein encapsidates newly synthesized viral RNA to form nucleocapsid cores in the cytoplasm. The E3 protein is translated after the capsid protein, and contains the signal sequence that directs the remainder of the polyprotein translation to the ER membranes (Figure 1B,C). The polyprotein traverses the ER membrane several times. The E2 protein is translated with two transmembrane domains, containing the translocon signal for 6K. The 6K protein is thought to have two transmembrane helices connected by a short cytoplasmic loop (Figure 1B). The second helix in 6K is the translocation signal for E1, usually the last protein to be translated. The E1 protein has a single transmembrane domain, leaving a short cytoplasmic C-terminal tail. When frameshifting in the 6K gene occurs, the E3 and E2 proteins are produced as described above. However, rather than two transmembrane helices in 6K, the TF protein’s unique C-terminus is translated into the cytoplasm and no E1 is made (Figure 1C). Using radioisotope labeling, antibodies, and mass spectrometry to detect TF, it is estimated that frameshifting occurs <30% of the time [13,14,15,16]. The consequence of translating E2 but not E1 due to the frameshifting event is not understood.

After the structural proteins are translated, they undergo conformational maturation and localization to become assembly-competent. Cellular enzymes cleave the structural polyprotein into its individual constituents (Figure 1B,C). Signalase cleaves E1 from 6K, and E2 from 6K and TF [17,18]. As a result, the second transmembrane domain of E2 is released from the membrane into the cytoplasm. The E3 protein remains covalently attached to E2 and aids with cellular chaperones in the proper folding of E2 and E1 [19,20,21,22,23,24]. While in the ER, E3 + E2 (also called pE2 in the literature) and E1 associate into heterodimers. Those heterodimers trimerize into complexes called spikes [25,26]. Glycosylation moieties are added to E3, E2, and E1 and subsequently modified as the spikes transit through the secretory system to the plasma membrane. The cellular protease furin cleaves E3 from E2 in the *trans*-Golgi, making spikes fusion-competent [27,28].

The last step before particle release is budding. Some enveloped viruses, e.g., human immunodeficiency virus I (HIV-I), require canonical host cell machinery (such as ESCRT—endosomal sorting complexes required for transport) to bud from cells [29,30]. Other enveloped viruses such as influenza encode their own budding protein [31,32]. Alphaviruses use an ESCRT-independent mechanism for budding, but it is not known if other host proteins are required or if a viral protein aids in budding [31,32]. Two models proposed for alphavirus budding are: (1) budding is driven by an association between viral glycoproteins followed by interactions with the capsid and RNA, and (2) preformed nucleocapsid cores drive interactions between glycoproteins that result in budding. Specifically, it has been shown that three amino acid residues in the cytoplasmic tail of E2 (residues 400–402 in Sindbis) interact with a surface pocket on the capsid [33,34,35]. In multiple cell types, preformed cytoplasmic cores are observed interacting with spikes at interior sites, sometimes called cytopathic vacuoles (CPV-II) [36,37]. While the role of CPV-IIs in viral release is still being investigated, they are hypothesized to represent transport intermediates for viral components to the site of budding. In arthropod cells, budding is observed at internal membranes, while this usually occurs at the plasma membrane from vertebrate cells [37,38,39]. The mechanism of alphavirus budding is not known but studies (see below) have attributed defects in assembly and budding to mutations in the *6K* gene.

## 3. Enter TF, the Other 6K

In 2008, Firth and colleagues bioinformatically predicted and experimentally demonstrated that the *6K* gene actually produces two distinct protein products, 6K and TF [14]. This occurred via a (−1) ribosomal frameshift site that is highly conserved across the alphaviruses. Indeed, all the sequenced alphavirus genomes to date contain a heptanucleotide slip site that is essential for the production of TF [14,40,41,42], and TF production has been demonstrated experimentally in Semliki Forest (SFV), Sindbis (SINV), Chikungunya (CHIKV), and Salmon pancreatic disease virus [14,15,16,43]. In Venezuelan equine encephalitis virus (VEEV), natural variants missing portions of 6K, often losing the TF slip site, were detected by deep sequencing [44]. Additionally, among the fish alphavirus family, natural variants have been isolated with deletions in 6K [45]. The genomes with 6K deletions have been shown to recombine in animal hosts to reincorporate the *6K* gene when it is available from a heterologous template [43,46]. Overall though, the genetic capacity to produce the TF protein from the *6K* gene via frameshifting is nearly universally present among the alphaviruses.

Frameshifting in viruses is well-studied in the classic case of the retroviruses where the Pol open reading frame is in the (−1) reading frame relative to Gag upstream [47,48,49]. Many viruses use frameshifting as a strategy to maximize their coding potential (recently reviewed in [50,51]). What we understand for the alphaviruses is that as the ribosome translates the 6K message, it encounters a heptanucleotide slip site about two-thirds of the way through the gene, followed by a poorly defined spacer and then a stable secondary structure that varies between viruses [52,53]. One investigation used a dual luciferase reporter to measure the frameshifting rates induced by alphavirus signals that were predicted to fold into RNA secondary structures including stem loops, hairpins, and pseudoknots [52]. The dual luciferase reporter has allowed investigation into additional species beyond the prototype alphaviruses, including Middelburg, sleeping disease, southern elephant seal, and Getah viruses. The range reported for these disparate alphaviruses ranges from 1% to 48% [52]. Although the rates varied, in the New World alphaviruses VEEV, eastern equine encephalitis virus, and western equine encephalitis virus, the tandem stem loop stimulatory signals downstream were confirmed, and also found to not be controlled by miRNA [53]. At a low frequency, these signals can stimulate the ribosome to slip backwards by one nucleotide into the (−1) reading frame, and then translation continues in the new frame to produce the TF protein, resulting in a unique C-terminus from that of 6K. E1 is not translated when TF is produced.

**Figure 2:**
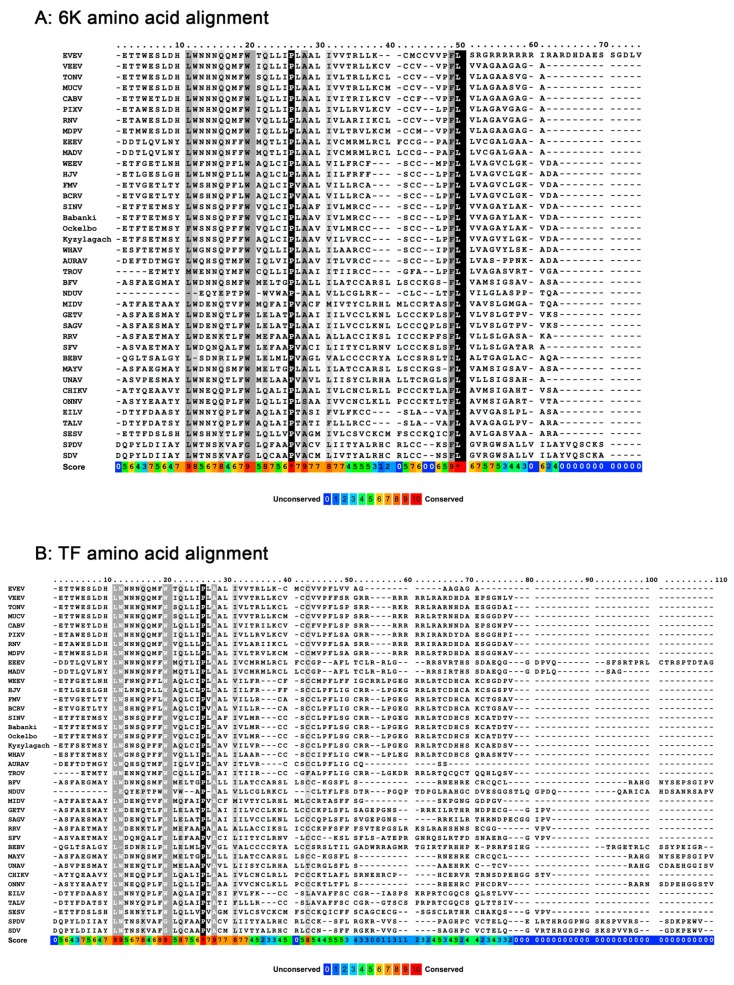
Multiple sequence alignment of (**A**) 6K and (**B**) TF with representatives from all International Committee on the Taxonomy of Viruses [57] recognized alphavirus species, and the newly discovered Taï Forest virus [42]. Sequences were retrieved from GenBank [58] with the accession numbers that follow. The 6K and TF translations were then aligned with the online PRALINE multiple sequence alignment program using the default parameters for homology-extended alignment [59,60,61,62]. The order in which the viruses are listed follows their relationships according to phylogeny, see [40]. The colored boxes across the bottom represent the consistency score (on a scale of 0–9 *), where blue is least conserved and red is most conserved. Scores of 8 or above are highlighted. Virus abbreviations and accession numbers are as follows: Everglades virus (AF075251) (EVEV); Venezuelan equine encephalitis virus (NC_001449) (VEEV); Tonate virus (AF075254) (TONV); Mucambo virus (AF075253) (MUCV); Cabassou virus (AF075259) (CABV); Pixuna virus (AF075256) (PIXV); Rio Negro virus (AF075258) (RNV); Mosso das Pedras virus (AF075257) (MDPV); eastern equine encephalitis virus (NC_003899) (EEEV); Madariaga virus (NC_023812) (MADV); western equine encephalitis virus (NC_003908) (WEEV); Highlands J virus (HM147988) (HJV); Fort Morgan virus (NC_013528) (FMV); Buggy Creek virus (HM147986) (BCRV); Sindbis virus (NC_001547) (SINV); SINV strain Babanki virus (HM14798); SINV strain Ockelbo virus (M69205); SINV strain Kyzylagach virus (AF339478); Whataroa virus (HM147993) (WHAV); Aura virus (NC_003900) (AURAV); Trocara virus (HM14799) (TROV); Barmah Forest virus (NC_001786) (BFV); Ndumu virus (HM147989) (NDUV); Middelburg virus (EF536323) (MIDV); Getah virus (NC_006558) (GETV); Sagiyama virus (AB032553) (SAGV); Ross River virus (DC5692) (RRV); Semliki Forest virus (NC_0032) (SFV); Bebaru virus (NC_016962) (BEBV); Mayaro virus (NC_003417) (MAYV); Una virus (AF339481) (UNAV); Chikungunya virus (YP_006491244) (CHIKV); O’nyong-nyong virus (M33999) (ONNV); Eilat virus (NC_018615) (EILV); Taï Forest virus (NC_032681) (TALV); southern elephant seal virus (HM147990) (SESV); salmon pancreas disease virus (AJ316244) (SPDV); sleeping disease virus (NC_003433) (SDV).

The 6K and TF proteins each contain an identical N-terminal transmembrane domain and a short cysteine-rich cytoplasmic loop prior to their unique C-terminal ends (Figure 2). As integral membrane proteins, 6K and TF are both found in membranous fractions of cells, and extract with anionic (SDS and sodium deoxycholate), nonionic (Nonidet™ P-40, IGEPAL^®^ CA-630, and Triton X-100), and zwitterionic (CHAPS-3-((3-cholamidopropyl) dimethylammonio)-1-propanesulfonate) detergents from eukaryotic and bacterial cells [14,16,43,54,55,56]. Apart from conserved hydrophobic membrane segments, the N-terminus also maintains several conserved residues (shown in Figure 2), and two or more cysteine residues prior to the slip site. The C-terminal domain of 6K is thought to be a second transmembrane domain, and the C-terminus of TF appears to be a hydrophilic cytoplasmic extension. The length of TF after the slip site varies by alphavirus from a predicted seven amino acids long in Aura virus to 46 amino acids long in the fish alphaviruses, and no striking conservation is noted.

Studies up to this point support that both the 6K and TF proteins are important in budding, despite being nonessential in tissue culture. Recombinant viruses not producing 6K and TF (∆6K), producing only 6K but not TF (6K-only, also called TF-null or ∆TF), and other TF mutants negatively impact infectious and total virus output from infected host cells compared to WT [13,15,55,63,64,65]. The absence of 6K and TF in ∆6K reduces virus production by 10–100 fold, and those virions also lack the structural integrity characteristic of WT particles [16,63]. TF is released in the virus particle while 6K remains primarily at interior cell membranes [14,15,16,66]. Despite differences in experimental setup and somewhat varying results for different alphaviruses, it is clear that whether concentrated in the perinuclear ER/Golgi regions or the plasma membrane, 6K and TF localize differently from each other [13,14,15,16,66]. Thus, the 6K and TF proteins affect released virus particles by functioning inside the host cell where budding is initiated.

Several functions attributed to the 6K protein have been experimentally determined. We will discuss these results in light of 6K and TF both being present during an infection. In addition, we will discuss recent studies on the TF protein and summarize how these contribute to our understanding of virus assembly.

## 4. Looking Back at the Discovery of the 6K Protein

The structural polyprotein’s topology and orientation are critical for proper protein processing and function (Figure 1). Researchers studying the glycoproteins E2 and E1 were searching for the signal sequences responsible for their final membrane orientation detected at the surface of the virus. Studies in SINV and SFV quickly identified E3 as the insertion signal for E2 [67,68,69]. However, the signal for E1 had not yet been ascertained. E1 was a known Type I membrane protein, having its N-terminus in the ER lumen and a short cytoplasmic tail following its transmembrane anchor domain [4,70,71]. Because the entire N-terminal ectodomain of E1 is found in the ER lumen, the protein immediately before it in the polyprotein would need to provide a start-transfer signal to translocate E1 into the ER lumen.

In 1979, a report by Welch and Sefton described two small polypeptides produced during SINV infection of vertebrate cells. In addition to the previously identified E3 protein, a new protein of 4200 daltons called 4.2K was thought to represent the cleaved signal sequence for a glycoprotein [54]. The 4.2K protein was not glycosylated, incorporated into virions, or released into the media. This same group then demonstrated an analogous protein of 6000 Da was produced during SFV infection, and so named it 6K for 6 kDa [72]. Like the SINV 4.2K protein, the SFV 6K protein was not glycosylated, present in mature virions, or released into the media. The authors concluded that the SINV 4.2K and SFV 6K proteins were analogous, and the field now refers to the 6K gene product as the 6K protein. N-terminal peptide sequencing suggested that the gene encoding 6K was after E2 in the viral genome [73], and full-genome sequencing placed it unambiguously before E1 in the structural open reading frame [17,74]. When C-terminal segments of 6K were shown to allow heterologous E1 translation, it strengthened the idea that 6K functioned as the signal sequence for E1 [75,76].

The E2/6K and 6K/E1 junctions are cleaved by a host protease called signalase [11,77]. Signalase, a peptidase found in the ER lumen, was earlier hypothesized as one of two host proteins to process the alphavirus structural polyprotein [17,18]. Combined with a start transfer sequence found in the C-terminus of E2, this meant that both the N- and C-termini of 6K were positioned in the ER lumen [77]. We now also know that when 6K is deleted, the second hydrophobic stretch in E2 can serve as the start-transfer signal for E1, just as it does for 6K in a WT background [16,63,64].

How E1 translocates across the ER membrane into the lumen becomes irrelevant when TF is translated, however, because no E1 is translated. The lack of E1 production upon TF translation is interesting because there is no longer a 1:1 ratio between all the proteins encoded on the structural polyprotein. Various reports have suggested that in different cell lines, E1 and E2 are only stable and only make it to the cell surface if they associate in a 1:1 heterodimeric complex [78,79]. Low rates of TF translation could reflect that only a very small amount of TF is needed for its function. Low frameshifting rates could also reflect a need for maintaining a high amount of E1 translation for optimal spike assembly and low E2 misfolding levels.

## 5. Posttranslational Modification in TF, but not 6K

With both 6K termini in the ER lumen, it was immediately noticed that the short cytoplasmic connector loop was rich in cysteine residues. In the years before that, virus protein acylation, the covalent attachment of fatty acids to cysteine residues in proteins, had been demonstrated in alphavirus and rhabdovirus proteins [80,81,82]. Having seen that both E2 and E1 were modified by palmitoylation on cysteine residues [83,84,85], researchers next looked at the modifications on the 6K protein.

An antibody generated against an amino acid sequence shared by the N-termini of both 6K and TF immunoprecipitated two proteins from cells infected with SINV, one at 6 kDa (most likely TF) and one at 4.2 kDa (most likely 6K) [86]. Only the upper band was palmitoylated and budded into virions. From their experiments, they interpreted the results to mean their two bands were the same protein with different modification states. Phosphorylation and glycosylation were not present so the two bands were thought to represent alternatively palmitoylated 6K proteins [72,86]. Their conclusions were logical since 6K and TF have identical N-termini. Based on this paradigm, the 2008 publication on TF came as a surprise. Follow-up studies on TF were consistent with the earlier results. The TF protein (or larger 6K isoform), but not the 6K protein (the smaller 4.2K isoform), is palmitoylated on N-terminal cysteines [16] and is predominantly present in released virions [14,15,16,86].

## 6. Mutations that Control the Production of 6K and TF during an Infection

The *6K* became the first (and only) alphavirus gene to allow the recovery of an infectious virus after its deletion, demonstrating that 6K is nonessential in tissue culture [63]. The ∆6K virions were more sensitive to thermal inactivation, but entry processes were not significantly altered compared to WT [64,87]. This did not, however, relegate 6K as uninteresting because the ∆6K virus manifested budding defects. Transmission electron microscopy on ∆6K-infected hamster cells revealed cores lined up beneath the plasma membrane, but very few budding particles, in contrast to the many budding particles seen in cells infected with WT. Additionally, the ∆6K virus yield from mammalian cell cultures is lower than WT at high growth temperatures [64]. These experiments support an accessory role for 6K and/or TF during spike maturation, where they are not required for budding but enhance assembly, perhaps by stabilizing spikes.

In another study, researchers found a deletion in the *6K* gene had consequences for glycoprotein processing. Sanz et al. generated del-6K, a partial internal deletion from residues 24 to 45 in 6K, which ablates the frameshift site required for TF production and acts functionally as a clean ∆6K mutant [88]. The del-6K mutant was also found to have impaired glycoprotein processing, perhaps resulting from misfolding and improper spike assembly. A revertant found to alleviate the defect in del-6K was a single amino acid change, Q21L in 6K. These researchers attempted, unsuccessfully, to complement the partial deletion with a WT 6K, although the authors cautioned that having the protein out of context was an important caveat to their results.

In contrast, recent reports from salmonid alphaviruses (SAV) have shown that a ∆6K virus is nonviable [43]. The SAV ∆6K defect appears to be due to inappropriate glycoprotein processing and/or trafficking. Maturation of the E2 protein in SAV is temperature-sensitive in fish and other cell line cultures, and this sensitivity of E2 may be reflected in the failure to recover infectious virus in ∆6K [89,90]. While in mammalian cells heterologous 6K or TF expression does not rescue a deficiency, in SAV-infected cells the helper plasmid 6K expression does rescue virus infection [15,43,88].

To analyze 6K and TF contributions to budding separately, various groups have utilized a virus with several silent mutations in the conserved slip site region that abrogate frameshifting and produce only the 6K protein. The 6K-only virus manifests a consistent delay in growth kinetics, but ultimately similar infectivity compared to WT is observed [15,16,53]. Since the 6K-only virus fares similarly to WT in tissue culture, it seems unlikely that TF is as critical for glycoprotein maturation as 6K. Furthermore, although TF is present in the WT virion, the published data do not support a spacer role for TF in the virion. TF protein remaining in the infected cell may function to exclude host proteins, corral spikes into the optimal budding conformation, or even interact with 6K.

## 7. Evidence that 6K Functions as a Spacer during Spike Assembly

The 6K spacer hypothesis was proposed shortly after a full SFV genome clone was constructed and made available [91]. Cysteine mutants in the N-terminus of 6K (residues also present in TF) display budding defects, with both reduced virus release and altered morphology. The 6K/TF cysteine mutants bud multi-cored particles with multi-hit kinetics of thermal inactivation [13,55,92]. This multi-cored defect was also observed for a temperature-sensitive mutation in SINV E2 [93]. Connecting the similarity in phenotypes with the unpublished observation that a lysine in the 6K protein can be crosslinked to the E2 tail cited in their paper, these authors postulated that an E2:6K protein interaction might be necessary for the E2 tail to interact with the capsid protein during budding. The cysteine 6K mutants and a lysine mutant in 6K were analyzed side by side with E2 mutations, and thus dawned the idea that 6K is a spacer. Under this model, 6K would aid in spike assembly and mediate the E2:CP interactions necessary for proper budding [55].

## 8. Direct Evidence for a 6K:E2 Interaction in Glycoprotein Maturation

Although a virus can bud without the 6K and TF proteins, there are reports showing that 6K interacts with the viral glycoproteins, perhaps prior to budding. In SFV, it was shown that 6K could be co-precipitated from infected cells with antibodies that pull out heterodimer spike complexes, and surface-exposed glycoprotein complexes also co-precipitated 6K [94]. Subsequent studies have, however, supported a predominantly internal localization for 6K [14,15,16,66,86]. One study using electron microscopy has reported that 6K may interact with intermediate filaments [95]. These experiments suggest a role for 6K during or after spike transport to the plasma membrane.

How could the 6K protein influence budding at the plasma membrane so dramatically from inside the cell? To investigate a potential direct interaction between the glycoproteins and 6K, Yao et al. used recombinant chimeric viruses that swapped in the Ross River virus (RRV) *6K* gene, or the *6K*:*E1* gene pair, with the SINV genes in a SINV backbone [96]. The chimeric construct that swapped in *6K* alone, and keeping both E2 and E1 as a SINV sequence, would have a native RRV 6K, but for TF, a RRV sequence in the N-terminus and SINV sequence in the C-terminus. This mutant virus had decreased budding and low E1 levels detected at the plasma membrane. The RRV *6K*:*E1* pair chimera in SINV, which would produce native RRV 6K and TF, was severely reduced in infectivity. Because E2 did not properly traffic to the plasma membrane, they proposed a sequence-specific interaction of 6K with the glycoproteins to get them folded properly and to the plasma membrane [96]. However, it is known that SINV and RRV glycoproteins are not conserved enough that they can be exchanged and function normally without sequence adaptation [97,98]. More evidence for 6K interacting with the glycoprotein transmembrane domains, in particular E2, came when a 39-amino-acid insertion into the RRV *6K* gene of the RRV 6K in SINV chimera was isolated. The insertion, in combination with other mutations, recovered titer, while the addition of individual E2 point mutants within the transmembrane domain reduced the chimera’s titer [99].

Additional studies on the E2 protein provided more evidence for direct interactions. London et al. screened for viable places to insert heterologous genes within the SINV E2 protein using a 13-amino-acid epitope [100]. During that process, they identified an insertion at residue 29 in the first transmembrane domain of 6K (6K29) [101]. The 6K29 mutant virions were indistinguishable from WT except that in the particle, they could not detect 6K (which we now know is the TF protein). Further, 6K29 was defective in host cell shutoff and glycoprotein processing was incomplete. Although entry steps were unaffected, they detected a trans-dominant negative effect on titer output in co-infections with WT [101]. In a follow-up study, they isolated a 6K29 revertant that showed increased neurovirulence [65]. The revertant contained two changes in the ectodomain of E2 (Q55H and H333Q). Further directed mutagenesis in the region corresponding to the transmembrane domain, specifically 6K A28R, generated mutants defective in glycoprotein processing. A change of Ala 28 to Met corrected glycoprotein processing, but not particle budding defects. The multi-cored defect was alleviated by the addition of the Q55H and H333Q E2 mutations [92]. Now that the TF protein has been identified, it is important to note that the insertions and other mutants isolated in these studies are in the shared N-terminus and it is not possible to rule out effects from TF mutation here.

How would a potential interaction between 6K and E2 affect particle budding? Since spike maturation is less efficient in 6K mutants, particle release is compromised. Consistent with this observation, results from several studies have reported higher cell-associated virus titers for the SINV 6K29 strain and the ∆6K virus in mammalian cells infected with SFV and RRV compared to WT [64,66,101]. The amount of cell-associated infectious virus reported in those studies can account for and sometimes exceed the reduction in media titer measured for 6K mutant infections compared to WT. The cell-associated titer is hypothesized to represent intracellular budding or surface-associated virus. Internal budding is regularly observed for WT virus in mosquito cell infections [37,39]. These data suggest that a 6K deficiency may simply route virus budding to an alternate exit pathway that is less efficient at release from mammalian cells. These functional budding defects in the ∆6K virus and the known effects on glycoprotein processing support the idea that an interaction between the glycoproteins and 6K is important for budding.

## 9. 6K is a Viroporin

Before TF was discovered, 6K was also known to have a role beyond direct interactions with spike proteins to promote effective spike maturation as a modulator of membrane permeability. The 6K protein is classified as a virus-derived ion channel, or viroporin [102]. Viroporins are usually small proteins with hydrophobic stretches that oligomerize to form channels primarily comprised of alpha-helices [103]. Viroporins self-assemble and often demonstrate ion selectivity. Early on, hydrophobicity plots of the 6K protein were noted to resemble those of other small virus proteins that modified membrane permeability [104]. Indeed, when the SINV *6K* and *TF* genes are overexpressed alone in bacterial cells, they cause lysis [15,105]. Various studies have demonstrated that 6K overexpression in bacterial cells also increases permeability to larger compounds such as the hydrophilic aminoglycoside hygromycin B (MW 527) [106,107,108,109]. Both synthetic peptides and recombinantly expressed full-length 6K protein from RRV and Barmah Forest virus promote ion flow when added to planar membranes in vitro [56]. Enhanced ion flow across cell membranes was also observed when 6K mRNAs capable of producing truncated TF products were injected into amphibian oocytes [110]. In planar lipid bilayers, the 6K peptides demonstrate a selectivity towards cations, preferring monovalent to divalent cations. In oocytes, translated products increased cytosolic calcium levels and activated endogenous potassium chloride efflux. These expression system experiments support intrinsic ion channel activity of the 6K protein. In contrast, for TF, the only data currently published demonstrates cell lysis upon overexpression in bacterial cells [15]. However, most of these experiments using the cloned *6K* gene would have likely translated TF truncation products. Therefore, additional studies looking independently at TF activity as an ion channel or pore would be informative.

In addition to demonstrating that a viral protein possesses stimulatory or intrinsic ion channel activity, the rigorous establishment of any viral protein as a functional viroporin uses evidence from appropriate stages during the virus life cycle [111]. A series of mutations and rescue attempts provide mounting evidence to support 6K’s role in membrane permeabilization in standard cell culture models. The *6K* gene expressed from a viral genome, both with and without a translocation start signal to provide proper protein orientation, is the only major contributor to the membrane permeability changes observed in eukaryotic cells with normal intracellular trafficking [107]. Mutations both within the 6K transmembrane domain and at conserved bulky hydrophobic residues before the first transmembrane domain demonstrated that these regions are important for the membrane-permeabilizing phenotypes of 6K [56,106]. Specific residues in the N-terminus and within the transmembrane domain affecting SINV 6K ion channel activity are also critical for the formation of intracellular virus structures and normal spike transport [112]. Although 6K is sufficient to induce membrane permeability, the E1 transmembrane domain and residues immediately preceding it have also been shown to induce membrane permeability [113]. These data are the foundation for understanding the mechanism by which 6K host ion permeability influences the outcome of a viral infection, and we can relate what is now known about 6K to other viroporins to formulate guiding hypotheses.

Studies on other known viroporins highlight the mechanisms by which membrane permeability to ions influences the outcome of virus infection. The state of this field for viroporins that modulate intracellular ion homeostasis to affect pathogenesis and specifically promote virus release including influenza A virus M2 protein, hepatitis C virus p7 protein, coronavirus E protein, and HIV-1 Vpu protein has been reviewed in detail [103,114,115,116]. Here we mention briefly two proteins, M2 and p7, whose viroporin activity and mechanism of action in the virus lifecycle are well documented. The influenza A M2 protein is found in the virion and functions during both entry and virus release from the host cell. Infectious virus release is affected by M2 at the levels of pH control in the secretory system and direct alteration of membrane curvature [117,118,119]. Structures for the homotetrameric M2 proton channel are available [120,121,122]. The hepatitis C virus p7 protein forms a hexameric complex [123,124]. The p7 ion channel exhibits low selectivity and is linked to enhanced viral release, potentially at the stage of glycoprotein maturation inside the cell [124,125,126,127]. The influenza A M2 protein ion channel activity is inhibited by the commercially licensed adamantane compounds [128,129]. Various groups are pursuing p7 as a target for anti-hepatitis C virus therapy (see for example, www.biotron.com.au trials with the BIT225 compound, universal trial number U1111-1150-4404) [124,130]. Interestingly, M2 [131,132] and coronavirus E protein [133] are also palmitoylated, and palmitoylation is known to regulate cellular ion channel activities [134]. There is evidence that 6K may be similar to these other viroporins. Defects observed in the SINV del-6K mutant, which does not produce normal 6K, can be partially rescued by expression of the full-length HIV-1 Vpu protein [88,135]. Substituting in only the Vpu transmembrane helix, or full-length p7, complements infectious particle production in infections without 6K present, and this activity is inhibitor-dependent [112]. Studies on TF ion channel activity during infection are not available. These data support a mechanisms of action for 6K that is similar to these other viroporins, where ion modulation ties directly to virus exit.

## 10. TF as a Virulence Determinant in Animal Infections

The TF protein in particular has been reported as a virulence factor by virtue of the fact that when it is missing, virulence is severely attenuated. This phenotype has been observed in several alphavirus infections. In SINV, 6K-only and TF mutants were used to infect a mouse model for neuropathogenesis [15]. All mice infected with TF mutants displayed reduced morbidity and increased survival compared to WT-infected mice. A similar outcome was seen with a CHIKV ∆6K virus, where symptoms were severely reduced in young infected mice, though the immune response was still adequate to protect the animal from future CHIKV challenge [136]. An RRV ∆6K strain is also suitable for vaccination and protection against challenge by other alphaviruses species in a mouse model [66]. These results may bring new relevance to the VEEV 6K deletions found to arise naturally during in vivo and tissue culture infections [44]. The deletions and point mutations reported all fell near or around the slip site required for TF production and tended to occur later in infection, which may function to modulate some important aspect of pathogenesis [44]. Those authors proposed that the slip site region could be a hotspot for recombination or template switching. This is consistent with the results of Kendra et al., showing mutations in 6K that prevent TF frameshifting significantly reduce morbidity and mortality in the same New World alphavirus in mice infected via aerosol [53]. From these results, Kendra et al. hypothesized that TF may be needed for passage across the blood–brain barrier or neuroinvasiveness [53].

A related phenomenon is observed in the alphaviruses that infect fish. The fish alphaviruses are currently causing a severe burden on farmed salmonids in northern Europe and elsewhere [137]. The SAV ∆6K actually is not viable in cell culture and recombination can occur in both cell culture and in injected fish that yield infectious virus with at least partial 6K sequences capable of causing pathology in subsequent hosts [43,46]. Indeed, when first isolating SAV, Weston and colleagues initially reported a small *6K* gene, but later found that *6K* simply had deletion variants [138,139].

While no direct mechanism is known, mutations in the *6K* gene have also been reported in alphavirus strains causing outbreaks worldwide. Most lineage-defining mutations are found in the E1 and E2 glycoproteins; however, a few reports also identify mutations in the *6K* gene. No work has yet addressed TF production in circulating strains. In fish alphaviruses, there is little sequence variation in full SAV *6K* genes, but the isoleucine at position 7 is mutated to threonine [140]. In human epidemic strains there have also been a few *6K* variations reported. For example, the CHIKV strain in Mexico of the Caribbean lineage (Asian genotype) was found to sustain the 6K L20M mutation [141]. A set of isolates from Colombia contained the same mutation, as well as additional mutations in the C-terminus of 6K that would also affect the TF sequence [142]. Interestingly, most subunit and virus-like particle vaccination approaches retain the *6K* gene sequence [143,144]. Although the reasons often given for this are that E2 requires E1 for proper folding and presentation on the cell surface, that is not always the case. Overall, variations in the *6K* and *TF* gene sequences isolated from the environment may provide useful leads to researchers pursuing studies in TF virulence.

## 11. Future Directions in 6K and TF Research

Very little new information on the mechanism by which 6K functions to affect budding or other viral processes has become available since the discovery of the TF protein, even though we now know that many of the budding phenotypes originally identified for 6K are more likely attributable to TF. Neither 6K nor TF are required for virus propagation in cell culture but the TF protein from several viruses has been shown to be a virulence factor. For this reason, it is of significant interest to uncover the independent functions of 6K versus TF during an alphavirus infection. Below we discuss research areas that are critical to understanding the functional roles of the 6K and TF proteins.

### 11.1. How Does 6K Promote Budding from Inside the Cell?

**Figure 3:**
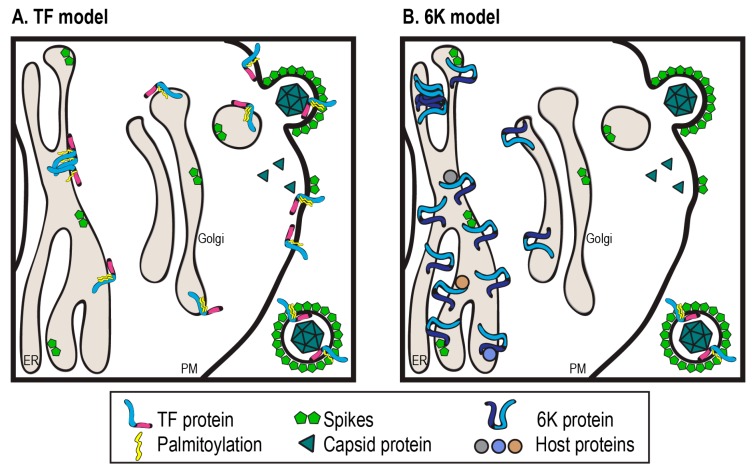
Model representing our current understanding of TF and 6K. (**A**) TF is less abundant than 6K. It is palmitoylated and traffics from the ER to the plasma membrane (PM) where it is budded into virions. TF may oligomerize and function as an ion channel. (**B**) 6K is found concentrated at interior membranes where it likely interacts with the viral glycoproteins. The 6K channel properties resulting from oligomerization probably affect the ER. Additionally, 6K may interact with host proteins.

With 6K residing primarily at interior cellular membranes (Figure 3), the questions about 6K that need to be addressed center on its interactions with other proteins, viral and host, that serve to promote budding. Follow-up work on the interaction between 6K and E2 is needed, since disruption of spike maturation can directly lead to reduced productive viral release. Currently available data can be interpreted to fit the 6K spacer model, where 6K is not required for trafficking the glycoproteins to the plasma membrane, but defects in spike folding or oligomerization efficiency in the absence of 6K are inhibitory to effective budding. Secondly, it is of interest to determine whether 6K interacts directly with host proteins. Such an interaction could serve to alleviate host cell stress responses that might prevent budding, or conversely promote an environment optimal for budding. Since 6K mutants have increased cell-associated titer, studies to identify 6K interaction partners might also provide insight into the differences in the location of budding observed between infected arthropod and mammalian host cells. Studies on 6K can now be facilitated by using mutants in the frameshift slip site which do not produce TF.

### 11.2. What Regulates TF Translation and Palmitoylation?

While TF has been demonstrated to play a role in virus budding (Figure 3), its function in released virions remains unclear, and additional questions about the TF production and modification status remain unanswered. With regards to TF frameshifting levels, there is no quantitative data tracking TF levels within an infected cell population over time. Does the amount of TF increase linearly over time, or does a switch turn on TF in response to a specific cue? Plasmid-based frameshifting assays have allowed the dissection of the contributions that the RNA itself has to stimulate frameshifting, but we do not know if there are any proteinaceous factors that influence frameshifting. Understanding this mechanism in different alphaviruses has good promise for safe vaccine strain design and potentially the treatment of alphavirus diseases [53]. In addition to regulating the frameshifting rates by secondary structure elements, natural variants with losses of TF frameshifting elements may be another way to control infection in vivo [44,46]. Is this hotspot of recombination a way to control pathogenicity? As it is, we have limited data about TF levels and function in different host cell types, and in different alphavirus species, and no mechanistic information outside the standard lab cell lines and animal models.

The only known posttranslational modification on TF is its palmitoylation. It is intriguing that in WT, the TF protein is always modified, at least in SINV where the studies have been done. We do not, however, have any sense for whether the modification on TF is dynamic. Related to that, it is also unknown which cellular palmitoyl transferases actually modify TF. The palmitoylation studies on TF thus far have focused narrowly on vertebrate cell hosts, but it is of interest to characterize palmitoylation in mosquito cells as well. TF trafficking is linked to palmitoylation status, but there is also evidence that the transmembrane domain shared by 6K and TF may interact specifically with E2’s transmembrane domain. What is the nature of such an interaction, and what impact does it have on not only protein trafficking to the plasma membrane, but also budding?

Studies focusing on TF alone are challenging without a TF-only virus. Constructing such a mutant is complicated by the need to ensure all the viral proteins in the polyprotein have the correct final membrane orientation. For example, attempting to generate a clone with the order CP-E3-E2-TF-E1 is not possible because the E1 ectodomain would be in the cytoplasm rather than the lumen. Also, expressing TF after E1 would result in a TF protein with its N-terminus in the cytoplasm rather than in the lumen. A clever strategy using inert linkers or artificial signal sequences is therefore needed to build this useful virus tool.

### 11.3. What is the Structure and Oligomeric State of the 6K and TF Proteins?

Our knowledge of the structure of 6K and TF is based on secondary structure predictions and cleavage of the structural polyprotein. In order to determine how 6K and TF each affect particle release, it is important to determine the structure of the individual proteins, their functional oligomeric state, and where and how TF is organized in the released virion. Knowing the structure of the individual proteins in the host and in the released virion would provide a structure-function link to identify the what and how of 6K and TF function. No experimentally determined structures for these proteins exist, and neither has been resolved in whole virus [145,146,147,148,149,150]. Our view on their membrane topology is based mainly on the known topology of the pre-cleavage structural polyprotein, where the termini must be in the ER for signalase to cleave, and the hydrophobic properties of the primary sequence. The 6K and TF proteins are translated with identical N-termini and unique C-termini. TF is palmitoylated at multiple cysteine residues in the N-terminus. Though the N-terminal cysteines are in both proteins, 6K is not palmitoylated. The precise membrane topology of the C-terminus of 6K is unknown. It likely is translated as a two-pass protein with both termini in the ER lumen and available for cleavage by signalase, but post-cleavage it has been proposed to adopt a Type I membrane protein (single pass) or hairpin conformation [11,73,77,92,106]. Kyte-Doolittle and other hydrophobicity profiles support two transmembrane domains in 6K [43,56,88,106]. However, the predicted transmembrane domains may represent a single integral membrane helix. If 6K folds into a single membrane-spanning helix, its cysteine residues could reside in the membrane rather than in the cytoplasm, and this might explain why it is not palmitoylated on residues that are modified in TF. In comparison, even less is known regarding TF’s conformation, and its final membrane conformation has broad implications for its function. Different conformations for the two proteins could explain why TF and not 6K is palmitoylated on shared cysteine residues and how TF localizes to the plasma membrane in a palmitoylation-dependent manner in SINV [16].

The precise membrane insertion of small membrane proteins is often difficult to assess with confidence and there may be more than one correct answer, as seen for the coronavirus E protein. Evidence has been presented, suggesting that the coronavirus E protein adopts multiple membrane-spanning conformations, and a hairpin state [151,152,153]. These various orientations and different oligomeric states manifest clearly separable phenotypes [151,153]. Further study into the structure of the 6K and TF proteins may reveal a similar case.

Questions of immediate interest regarding TF relate to its presence in the virion. Is its presence incidental to some unknown function it carries out near sites of budding within the originator host cell? Do the low levels of TF in the virion serve an important purpose? How are the low copy numbers of TF organized in the virion? It is not known if the population of virus particles is homogenous in its TF content, though it is generally assumed so. Mutants missing TF, or lacking palmitoylation, are known to differ from WT in gross morphology and thermolability [13,16,64]. This is useful to know because it may shed light on how TF is selectively incorporated into the virus particle. Since 6K has also been reported to incorporate into SINV particles [15], the isoform that is incorporated may differ between alphaviruses based on localization.

### 11.4. Can We Target the Budding Proteins 6K and TF for Antivirals?

Very few antivirals on the market today are directed at virus escape. One well-known example is Tamiflu which blocks the neuraminidase activity required for influenza virus exit [154,155]. Viroporins often affect exit and have also been proposed as viable antiviral targets. In hepatitis C virus, inhibitors targeting the viroporin protein p7 are currently being tested in human trials [124,130]. With the widespread references to 6K’s role as a viroporin, both the 6K and TF proteins represent interesting potential targets for antivirals inhibiting the alphavirus exit process. We know that the involvement of 6K and TF in the spread of virus particles between permissive cells within an organism impacts disease severity. Preventing alphaviruses from exiting host cells and traversing to the next cell may therefore mitigate or prevent disease progression. More careful work is needed to demonstrate the importance of 6K specifically (without the potential for generating TF truncations), and TF also, in ion transport. In particular, studies addressing the effects of modulating ion channel activity in infected cells on virus production are high priority. Despite feasibility as an effective antiviral strategy, antivirals against virus release are not in widespread use because the details involved in virus escape from infected host cells are not well understood. Understanding the mechanisms of action of 6K and TF in escape could guide future efforts to fatally stall budding.

## 12. Conclusions

The story of 6K and TF is a classic scientific paradigm shift. For 20 years, studies on 6K were interpreted in the context of all the information available at the time, where 6K was a small membrane protein involved in budding. When TF was discovered, the old literature had to be re-interpreted in the light of a new paradigm. The *6K* gene is translated into two proteins that have separate roles in budding. Now, we know that 6K likely promotes virus release by its role inside the cell, including interactions with the glycoprotein spikes. The TF protein is present in virions and its function there remains unknown, but it is important for spread in an animal host. The road ahead is now populated with a new and exciting series of research questions whose answers will lead us to a better understanding of 6K and TF as individual proteins, and ultimately a better picture of enveloped virus budding.
